# The quality of migrant patients’ primary healthcare experiences and patient-centered medical home achievement by community health centers: results from the China greater bay area study

**DOI:** 10.1186/s12939-023-01929-z

**Published:** 2023-06-07

**Authors:** Yongjun Huo, Xun Kang, Chenyang Zhong, Leiyu Shi, Ruqing Liu, Ruwei Hu

**Affiliations:** 1 Department of Health Management, Sun Yat-Sen University School of Public Health, Guangzhou, Guangdong China; 2 The Third People’s Hospital of Foshan, Foshan Mental Health Center, Foshan, Guangdong China; 3 Sun Yat-Sen University School of Public Health, Guangzhou, Guangdong China; 4 John Hopkins School of Public Health, Baltimore, MD USA; 5 Guangdong Provincial Engineering Technology Research Center of Environmental Pollution and Health Risk Assessment, Department of Occupational and Environmental Health, School of Public Health, Sun Yat-Sen University, Guangzhou, China

**Keywords:** Migrant, Patient experiences, Primary healthcare, National Committee for Quality Assurance Patient-Centered Medical Home, Primary Care Assessment Tools

## Abstract

**Background:**

In China, Community Health Centers (CHCs) provide primary healthcare (PHC); however, few studies have examined the quality of PHC services experienced by migrant patients. We examined the potential association between the quality of migrant patients’ PHC experiences and the achievement of Patient-Centered Medical Home by CHCs in China.

**Methods:**

Between August 2019 and September 2021, 482 migrant patients were recruited from ten CHCs in China’s Greater Bay Area. We evaluated CHC service quality using the National Committee for Quality Assurance Patient-Centered Medical Home (NCQA-PCMH) questionnaire. We additionally assessed the quality of migrant patients’ PHC experiences using the Primary Care Assessment Tools (PCAT). General linear models (GLM) were used to examine the association between the quality of migrant patients’ PHC experiences and the achievement of PCMH by CHCs, adjusting for covariates.

**Results:**

The recruited CHCs performed poorly on PCMH1, Patient-Centered Access (7.2 ± 2.0), and PCMH2, Team-Based Care (7.4 ± 2.5). Similarly, migrant patients assigned low scores to PCAT dimension C—First-contact care—which assesses access (2.98 ± 0.03), and D—Ongoing care (2.89 ± 0.03). On the other hand, higher-quality CHCs were significantly associated with higher total and dimensional PCAT scores, except for dimensions B and J. For example, the total PCAT score increased by 0.11 (95% CI: 0.07–0.16) with each increase of CHC PCMH level. We additionally identified associations between older migrant patients (> 60 years) and total PCAT and dimension scores, except for dimension E. For instance, the average PCAT score for dimension C among older migrant patients increased by 0.42 (95% CI: 0.27–0.57) with each increase of CHC PCMH level. Among younger migrant patients, this dimension only increased by 0.09 (95% CI: 0.03–0.16).

**Conclusion:**

Migrant patients treated at higher-quality CHCs reported better PHC experiences. All observed associations were stronger for older migrants. Our results may inform future healthcare quality improvement studies that focus on the PHC service needs of migrant patients.

**Supplementary Information:**

The online version contains supplementary material available at 10.1186/s12939-023-01929-z.

## Background

In 2020, there were an estimated 281 million international migrants, comprising 3.6% of the world’s population [1]. In China in 2021, there were more than 376 million internal migrants [2]. Migrants experience considerable health inequity, defined as unjust and avoidable health differences that arise from socioeconomic discrimination or lack of access to health resources [3, 4]. For example, Guangzhou and Foshan, two modern metropolitan centers in South China’s Greater Bay Area, have attracted nearly 14.4 M internal migrants from other parts of China [5, 6]. Unfortunately, due to the rigid household registration system called “Hukou” and institutional discrimination in China, migrant patients face a higher disease burden and less access to appropriate and timely healthcare than urban or rural residents [7, 8].

Moreover, migrants often possess different sociodemographic characteristics than local residents, including differences in language, occupation, psychosocial characteristics, lifestyle, and consumption models. These differences affect migrant patients’ health status and often lead to health inequities [9].

Primary health care (PHC) mitigates health inequities by empowering individuals and communities and promoting social cohesion [10]. PHC service quality is critically important for health equity. High-quality PHC services should provide appropriate healthcare whenever needed, regardless of residency status. As indicated by the Plan of Health China 2030 and the Outline of the 14^th^ Five-Year Plan (2021–2025) for National Health, access to healthcare services (particularly PHC services) and health equity are the focus of many continuous healthcare quality improvement efforts [11, 12].

The 2009 Chinese health system reform attempted to establish a universal PHC delivery system to provide safe, effective, accessible, and affordable health services and encourage PHC providers to improve the quality of their patient-directed service [13]. Unfortunately, migrant patients and local residents have vastly different experiences when accessing Community Healthcare Centers (CHCs), the main PHC providers. We previously used the Primary Care Assessment Tool (PCAT) to evaluate the quality of patients’ experiences of PHC service [14]. Our results indicated that migrants had significantly worse PHC experiences than local residents, especially for first-contact utilization, ongoing care, family centeredness, community orientation, and cultural competence. There were also high levels of dissatisfaction, frustration and distrust in PHC service and General Practitioners (GP) among European migrants living in the UK [15]. And migrants is less satisfactory than the local population, especially in the attitudes of health workers and waiting times [16]. In terms of the factors that may affect migrants’ PHC service experiences, some studies noted several potential obstacles: differences in perceptions and expectations between GP and patients, the lack of communication and language skills of GP and cultural barriers [17, 18]. A study out of Guangzhou found that the experiences of rural-to-urban migrants relate to medical institution type and payment source [19].

According to Donabedian, the father of American quality management, quality can be defined as "structure-process-result" quality, and better process quality will bring better result quality [20]. “Process quality” and “outcome quality” can be reflected in providers’ and patients’ perspectives. Our previous study examined CHC service quality from patients’ and providers’ perspectives. Improved CHC service quality, as determined by the National Committee for Quality Assurance Patient-Centered Medical Home (NCQA-PCMH), improved the quality of patients’ PHC experiences, as determined by the PCAT [21]. Unfortunately, the effects of CHC service quality on migrant patients’ healthcare experiences remain unclear.

To help address this knowledge gap, we evaluated the achievement of PCMH by CHCs (an indication of institutional quality) using the NCQA-PCMH and evaluated the quality of migrant patients’ PHC experiences (an indication of individual quality) using the PCAT. Once again, we focused on CHCs in China’s Great Bay Area. We hypothesized that migrant patients treated at high-quality CHCs would report better PHC experiences. In other words, higher institutional PHC quality provision (as reflected in PCMH achievement) is reflected in higher individual PHC quality experiences.

## Methods

### Study setting and population

Our study was conducted within two major metropolitan areas in South China’s Greater Bay Area: Guangzhou and Foshan. We employed a multi-stage, stratified clustering sampling strategy in Guangzhou from August to October 2019 and in Foshan from August to September 2021. We selected four urban districts in Guangzhou (Yuexiu, Liwan, Haizhu, and Tianhe) and one in Foshan (Chancheng). As shown in Figure S1, ten urban CHCs were randomly selected: Linhua (LH) and Liede (LD) in Tianhe, Jianghai (JH) and Shayuan (SY) in Haizhu, HuangHuagang (HHG) in Yuexiu, Hualin (HL) in Liwan, Chancheng High-tech Zone Hospital (CHZH), Yong’an Hospital (YH), Nanzhuang Town the First People’s Hospital (NTFPH), and Foshan Fosun-Chancheng Hospital (FFCH) in Chancheng. Next, we randomly recruited one to three family physician groups from each CHC.

Participants were enrolled by the selected family physician group while visiting the CHC. Inclusion criteria were: age 18 years or older, patient’s household registered in other cities but patient residing in Guangzhou or Foshan for at least 6 months, and no auditory or visual impairments, mental illness, or compliance issues. All participants signed informed consent forms prior to any study-related procedures. The Human Studies Committee of Sun Yat-sen University approved the study’s protocol in compliance with the Declaration of Helsinki—Ethical Principles for Medical Research Involving Human Subjects (no. IRB2014.9).

### Assessment of the quality of migrant patients’ PHC experiences

We used the Primary Care Assessment Tool-Adult Short Version (PCAT-AS) to assess the quality of migrant patients’ PHC experiences. The Primary Care Policy Centre of Johns Hopkins University developed a series of PCAT scales with theoretical, practical, scientific, and objective advantages because scoring does not rely on respondents’ expectations, perceptions, or values but—rather—on patients’ real experiences [22]. The instrument is valid, reliable, and widely used in China and other countries [23–27]. The Chinese version of the original simplified PCAT has a reliability coefficient of 0.963, an acceptable test–retest reliability coefficient of 0.7 [21], and inclusion of various attributes of PHC [14, 21]. These include dimension B, first-contact utilization (the extent to which the primary care provider performs a gatekeeper function); C, first-contact access (whether patients can contact a physician in time when they need medical and health service); D, ongoing care (the continuous relationship between physicians and patients in primary care institutions); E, coordination of care (the interpersonal linkage of care among different levels of providers); F, coordination information systems (informational linkage of care through the use of an electronic information system); G, comprehensiveness of service available (the ability to perform a wide range PHC service); H, comprehensiveness of service provided (the appropriate provision of service during consultations by a PHC provider), and three derivative dimensions: I, Family Centeredness (the recognition of the family as a major participant in the diagnosis, treatment, and recovery of patients); J, Community Orientation (whether CHCs fully consider the needs of patients in the implementation of health service) and K, Cultural Competence (the provision of care that respects the beliefs, interpersonal styles, attitudes and behaviors of people as they influence health). Each dimension contained 3–5 items, totaling 36 items. A 4-point Likert-type scale was used to score each item, with “1” for “Definitely not,” “2” for “Probably not,” “3” for “Probably,” and “4” for “Definitely,” and “2.5” for “Not sure/Do not remember.” The average score of a dimension’s items comprised the dimension’s final score. The higher the PCAT score, the better the patient’s experiences and (presumably) the better the PHC service received.

### Assessment of CHC service quality

Since 2008, the National Committee for Quality Assurance (NCQA) has measured the quality of medical providers and practices. More than 10,000 practice sites and 50,000 clinicians have earned the NCQA-PCMH Recognition Seal [28]. NCQA-PCMH questionnaire responses may help identify service quality problems, reduce healthcare costs, and improve patients’ experiences and health [29]. Thus, to explore institutional service quality, we translated and adopted the 2014 NCQA-PCMH tool authorized by the NCQA website.

The 2014 NCQA-PCMH consists of 6 major panels, including PCMH1 “Patient-Centered Care Accessibility,” PCMH2 “Team Care,” PCMH3 “Population Health Management,” PCMH4 “Management and Support of Health Care,” PCMH5 “Care Coordination and Care Transitions,” and PCMH6 “Performance Measurement and Quality Improvement.” Each panel includes 3–7 elements, one of which was a required element (for a total of 27 elements) and 2–11 specific entries for each element, each including a key entry (for a total of 178 entries). There are separate scoring criteria and rules for each element under each of the instrument’s panels. Based on compliance with the criteria required for the different entries of each element, scored by the percentage for that element is obtained (if the key entry for that element was not met, the individual percentage for that element was 0%). Patients’ questionnaire responses were calculated to determine the total and panel scores. The maximum total score was 100, divided by three levels, with 35–59 indicating Level 1, 60–84 indicating Level 2, and 85–100 indicating Level 3 [30]. The higher a CHC’s NCQA-PCMH level, the better the quality of its PHC service [31].

### Covariates

Patients’ information, including sociodemographics, health status, and medical-related characteristics, was obtained via a self-report questionnaire. Sociodemographic information includes age (years), sex (male vs. female), education (junior high school or below vs. senior high school or above), and disposable income (DPI; ≤ 80,000 RMB vs. > 80,000 RMB). Health status was self-reported (fair/poor/very poor and excellent/good), and chronic health conditions were scored as present or not (i.e., no chronic disease vs. any chronic disease). Medical-related information consisted of medical insurance data (resident, employee, and business insurance) and the number of times CHCs were visited during the preceding year (≤ 5 vs. > 5).

### Data collection

Patients recruited from CHCs required 20–25 min to fill out the PCAT scale and the self-report questionnaire. The supervisors of the selected CHCs were invited to complete the NCQA-PCMH recognition questionnaires. If a patient or supervisor did not understand a questionnaire item, a well-trained investigator promptly offered an explanation, thus assuring the authenticity and reliability of the obtained data. The questionnaires were all inspected upon completion by an investigator to make sure that all questions were answered. If an item was missed or if the respondent did not understand the item, the response was immediately verified to ensure data quality.

### Statistical analyses

Continuous variables are presented as means ± standard deviations, and relative frequencies are calculated for categorical variables. We used Welch’s analysis of variance (ANOVA) to compare the PCAT scores at different CHCs and used the Games-Howell test for the subsequent multiple comparisons. We used a general linear model (GLM) to estimate associations between the PCAT and NCQA-PCMH. (1) represents the crude model; (2) signifies adjustments for age, sex, education, disposable income, self-reported health status, chronic health conditions, medical insurance type, and the number of visits to CHCs during the preceding year [14, 19, 32–35]. City was treated as a random effect.

We stratified the GLM by age to explore potential age-related effects. The results are presented as β values (95% CI). The statistical analyses were conducted using SPSS version 21.0 (SPSS Inc., Chicago, IL, USA) and R studio. All statistical tests were two-sided. P-values < 0.05 were considered statistically significant.

## Results

### CHC service quality

**Fig. 1:**
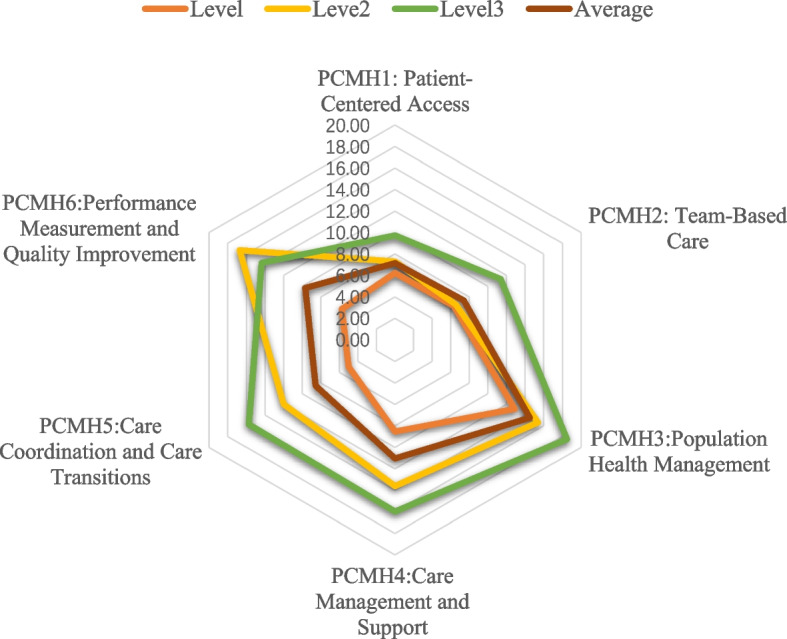
Average and sub-dimension scores of the National Committee for Quality Assurance Patient-Centered Medical Home (NCQA-PCMH)

The ten selected CHCs had an effective NCQA-PCMH response rate of 100%. As shown in Table S1 and Fig. 1, SY CHC in Guangzhou and FX CHC in Foshan had the best PHC service quality as suppliers (i.e., PCMH level 3); meanwhile, YA and GXQ CHCs in Foshan had the poorest service quality, with the scores lower than 35 (i.e., PCMH level 1’s cutoff point). Note that, for the purposes of this study, we categorized these CHCs as Level 1. In all, there were two Level 3 CHCs (SY and FX), two Level 2 CHCs (JH and HHG, both in Guangzhou), and six Level 1 CHCs (LH, LD, and LH in Guangzhou, and NZ, YA, and GXQ in Foshan). For all the sections, PCMH1, Patient-centered/Access (7.2 ± 2.0), and PCMH2, Team-based Care (7.4 ± 2.5) had the lowest average scores.

### Patients’ baseline characteristics

In all, 482 migrant patients were invited to complete the PCAT questionnaires. Ultimately, 473 were included for an effective response rate of 98.1%. Of the 473 participants, 374 were younger than 60 years (79.1%), and the majority (*n* = 248; 52.4%) were male. When stratified by NCQA-PCMH level, there were 289 Level 1, 51 Level 2, and 133 Level 3 CHCs. There were significant differences in sex, education, disposable income (RMB), self-rated health status, and chronic disease status among the patients seen be CHCs of different levels (*P* < 0.05; Table 1).

**Table 1:** Characteristics of migrant patients stratified by NCQA-PCMH Levels, n (%)

Characteristics	Level1 (*n* = 289)	Level2 (*n* = 51)	Level3 (*n* = 133)	Total (*n* = 473)	*P* value
Age(years)					0.08
≤ 60	222(76.8)	38(74.5)	114(85.7)	374(79.1)	
> 60	67(23.2)	13(25.5)	19(14.3)	99(20.9)	
Sex					< 0.001
Male	168(58.1)	16(31.4)	64(48.1)	248(52.4)	
Female	121(41.9)	35(68.6)	69(51.9)	225(47.6)	
Education					0.004
Junior high school or below	132(45.7)	29(56.9)	43(32.3)	204(43.1)	
Senior high school or above	157(54.3)	22(43.1)	90(67.7)	269(56.9)	
Disposable personal income (RMB)					0.005
≤ 80,000	152(52.6)	17(33.3)	80(60.2)	249(52.6)	
> 80,000	137(47.4)	34(66.7)	53(39.8)	224(47.4)	
Medical insurance					0.42
Employee	146(50.5)	28(54.9)	56(42.1)	230(48.6)	
Resident	114(39.5)	19(37.3)	64(48.1)	197(41.6)	
Business insurance	29(10.0)	4(7.8)	13(9.8)	46(9.7)	
Self-rated health status					0.003
Fair/Poor/Very poor	90(31.1)	27(52.9)	36(27.1)	153(32.3)	
Excellent/Good	199(68.9)	24(47.1)	97(72.9)	320(67.7)	
Chronic disease status					< 0.001
No chronic disease	182(63.0)	41(80.4)	108(81.2)	331(70.0)	
Any chronic disease	107(37.0)	10(19.6)	25(18.8)	142(30.0)	
Times of doctor visits in the last year					0.09
≤ 5	168(58.1)	30(58.8)	92(69.2)	290(61.3)	
> 5	121(41.9)	21(41.2)	41(30.8)	183(38.7)	

Data are n (%)

The level is determined by NCQA-PCMH

The P-value is based on the Chi-square test

### Evaluation of the quality of migrant patients’ PHC experiences

The migrant patients’ total average PCAT score was 3.12 ± 0.02. The dimensions with the lowest PCAT scores were C, First-contact care/access (2.98 ± 0.03), and D, ongoing care (2.89 ± 0.03). When stratified by NCQA-PCMH levels, the migrant patients at Level 3 CHCs had the highest PCAT total score and higher sub-dimension scores (*P* < 0.05; Table 2) reflecting that higher institutional quality is related to higher individual quality.

**Table 2:** migrant patients’ experiences determined by PCAT, stratified by NCQA-PCMH Levels (*n* = 473)

Primary care dimensions	Total *n* = 473	Level1 *n* = 289	Level2 *n* = 51	Level3 *n* = 133	*P* value
B First-contact in terms of utilization	3.16 ± 0.03	3.14 ± 0.04	3.06 ± 0.09	3.24 ± 0.05	0.16
C First-contact care in terms of access	2.98 ± 0.03	2.87 ± 0.04	2.74 ± 0.07	3.29 ± 0.04	< 0.001
D Ongoing care	2.89 ± 0.03	2.79 ± 0.04	2.65 ± 0.08	3.20 ± 0.05	< 0.001
E Coordination of care	3.17 ± 0.04	3.10 ± 0.06	2.95 ± 0.08	3.41 ± 0.06	< 0.001
F Coordination of information systems	3.33 ± 0.03	3.24 ± 0.04	3.32 ± 0.08	3.52 ± 0.04	< 0.001
G Comprehensiveness of service available	3.18 ± 0.03	3.04 ± 0.04	3.38 ± 0.05	3.40 ± 0.05	< 0.001
H Comprehensiveness of service provided	3.25 ± 0.03	3.19 ± 0.03	3.23 ± 0.07	3.38 ± 0.05	< 0.001
I Family centredness	3.20 ± 0.03	3.13 ± 0.04	2.94 ± 0.10	3.46 ± 0.04	< 0.001
J Community orientation	3.03 ± 0.03	2.97 ± 0.04	2.84 ± 0.08	3.23 ± 0.06	< 0.001
K Culturally competent care	3.11 ± 0.03	3.00 ± 0.04	3.20 ± 0.08	3.38 ± 0.05	< 0.001
Total score	3.12 ± 0.02	3.04 ± 0.03	3.03 ± 0.05	3.34 ± 0.04	< 0.001

*P* value is based on Welch’s analysis of variance (ANOVA)

*PCAT* The Primary Care Assessment Tool

### Associations between CHC PCMH achievement and the quality of migrant patients’ PHC experiences

Table 3 shows the associations between CHC PCMH achievement and patients’ PCAT scores. Adjusting for age, sex, education, disposable income, self-reported health status, chronic health conditions, medical insurance, and the number of visits to a CHC during the preceding year, PCAT scores were positively associated with CHC PCMH achievement (*P *< 0.05), except dimension B, First-contact/utilization, and dimension J, Community orientation. For example, the total PCAT score increased by 0.11(95%CI, 0.07, 0.16) with one level improvement in CHCs’ PCMH achievement.

**Table 3:** Associations between CHC PCMH achievement and migrant patients’ PCAT scores (*n* = 473)

	Crude*β*(95%CI)	*P*^a^	Adjusted^b^*β*(95%CI)	*P*^a^
Total	0.14(0.10,0.19)	< 0.001	0.11(0.07, 0.16)	< 0.001
B First-contact in terms of utilization	0.04(-0.02,0.11)	0.21	0.02(-0.05, 0.08)	0.6
C First-contact care in terms of access	0.19(0.13,0.25)	< 0.001	0.15(0.09, 0.21)	< 0.001
D Ongoing care	0.19(0.12,0.26)	< 0.001	0.16(0.10, 0.23)	< 0.001
E Coordination of care	0.14(0.05,0.22)	< 0.001	0.10(0.01, 0.18)	0.03
F Coordination of information systems	0.14(0.08,0.20)	< 0.001	0.12(0.06, 0.18)	< 0.001
G Comprehensiveness of service available	0.18(0.12,0.25)	< 0.001	0.18(0.12, 0.24)	< 0.001
H Comprehensiveness of service provided	0.09(0.03,0.15)	< 0.001	0.06(0.01, 0.12)	0.03
I Family centredness	0.15(0.08,0.21)	< 0.001	0.11(0.05, 0.17)	< 0.001
J Community orientation	0.12(0.05,0.18)	< 0.001	0.06(-0.01, 0.13)	0.06
K Culturally competent care	0.21(0.14,0.27)	< 0.001	0.17(0.10, 0.23)	< 0.001

*CHCs* Community health centers, *PCAT* The Primary Care Assessment Tool

^a^*P* value is based on the general linear model (GLM)

^b^Adjusting for age, sex, level of education, personal disposal income, self-reported health status and chronic health conditions, medical insurance and times of visiting CHCs last year, and city as a random effect

### Age as a potential modifier

Age was significantly associated with CHCs’ PCMH achievement. With the exception of dimension E—Coordination of care—migrant patients older than 60 years were associated with higher PCMH achievement (Table 4). For instance, the PCAT score for dimension C in older migrant patients increased by 0.42 (95% CI: 0.27–0.57) with each level of improvement in CHC PCMH status. Meanwhile, among younger migrants, the PCAT score for dimension C only increased by 0.09 (95% CI: 0.03–0.16).

**Table 4:** Association between CHC PCMH achievement and migrant patients’ PCAT scores by age (*n* = 473)

	β^a^	95%CI^a^	*P* value for interaction^b^
Total score			
≤ 60	0.06	0.01 ~ 0.11	< 0.01
> 60	0.40	0.29 ~ 0.51	
B First-contact in terms of utilization			
≤ 60	-0.03	-0.10 ~ 0.04	< 0.01
> 60	0.26	0.11 ~ 0.42	
C First-contact care in terms of access			
≤ 60	0.09	0.03 ~ 0.16	< 0.01
> 60	0.42	0.27 ~ 0.57	
D Ongoing care			
≤ 60	0.12	0.04 ~ 0.19	< 0.01
> 60	0.43	0.27 ~ 0.60	
E Coordination of care			
≤ 60	0.07	-0.03 ~ 0.17	0.35
> 60	0.20	0.03 ~ 0.38	
F Coordination of information systems			
≤ 60	0.05	-0.01 ~ 0.11	< 0.01
> 60	0.51	0.35 ~ 0.67	
G Comprehensiveness of service available			
≤ 60	0.13	0.07 ~ 0.20	< 0.01
> 60	0.33	0.18 ~ 0.49	
H Comprehensiveness of service provided			
≤ 60	-0.02	-0.08 ~ 0.04	< 0.01
> 60	0.46	0.33 ~ 0.60	
I Family centredness			
≤ 60	0.03	-0.03 ~ 0.09	< 0.01
> 60	0.44	0.28 ~ 0.60	
J Community orientation			
≤ 60	0.01	-0.06 ~ 0.08	< 0.01
> 60	0.26	0.08 ~ 0.43	
K Culturally competent care			
≤ 60	0.11	0.04, 0.18	< 0.01
> 60	0.55	0.39, 0.70	

*CHCs* Community health centers, *PCAT* Primary Care Assessment Tool

^a^After adjusting for sex, level of education, disposable income, self-reported health status, chronic health conditions, medical insurance, and the number of visits to CHCs during the preceding year; city was added as a random effect

^b^Calculated by adding an interaction item in the model

## Discussion

In the present study of 473 migrant patients, patients perceived PHC service quality to be better in higher-quality CHCs. All observed associations were stronger for migrants older than 60 years. To our best knowledge, this is the first report globally to explore the relationship between PCMH achievement by CHCs (institutional quality) and the quality of migrant patients’ PHC experiences (individual quality) service quality.

The structure–process–outcome model propounded by Donabedian states that better processes lead to better outcomes [20, 36]. In the present study, “process quality” reflected how CHCs administered PHC service. “Outcome quality” was reflected in the patients’ experiences. Thus, to improve patients’ experiences, CHCs must progress in their ability to provide PHC service.

The PCMH personalizes, prioritizes, and integrates PHC service to improve the health of individuals, families, communities, and the nation’s population by identifying and implementing new organizational practices and enhancing CHCs’ internal capabilities [37]. NCQA-PCMH recognition is important for assuring quality PHC service. The NCQA-PCMH can serve as a model when attempting to improve the quality of PHC service delivered by CHCs to migrant patients in China [29, 38].

In the present study, patients seen at Level 3 CHCs had higher PCAT and total sub-dimension scores compared to patients seen at Levels 1 or 2 CHCs. Furthermore, we found associations between PCMH achievement by CHCs and migrant patients’ PHC experiences, even after adjusting for confounders. Thus, the highest-level NCQA-PCMH CHCs provided the best care, consistent with the structure–process–outcome model proposed by Donabedian [20, 36].

Of the ten participating CHCs, six were NCQA-PCMH Level 1, the “worst” level. Level 1 CHCs may provide substandard care and require more improvement, especially in Patient-centered Access and Team-based Care. Similarly, migrant patients had the worst “First-contact” experiences pertaining to care access and ongoing care, consistent with Wu’s prior report [14].

Dimension D (“ongoing care”) was the lowest-scoring dimension. There are three types of continuity of care: informational, management, and relational [39]. Long-term relationships between physicians and patients develop over time. Migrants and other short-term residents may not have sufficient time to find and strengthen such relationships [40]. Most GPs in China are unfamiliar with migrants’ preferences, values, and backgrounds. This lack of familiarity is non-conducive to consistent management of long-term diseases. Team-based care models are better equipped to address health and social inequities [41]. The experiences of migrant patients accessing PHC can be improved by improving continuity-of-care in CHCs, as per PCMH 2 (“Team-based Care”) standards. To ensure ongoing demand for their available PHC service, CHCs should strive to provide personalized healthcare using a relatively fixed team of GP physicians. CHCs that create dynamic management systems will be better positioned to serve migrant patients, given their unique residency status [42].

Accessing PHC through CHCs was seen as difficult. This sentiment was reflected in the relatively low scores for PCAT C and PCMH 1. *Accessibility* refers to the ease with which a patient can converse with clinicians about any health issue (such as by telephone) and includes efforts to eliminate geographical, administrative, financial, cultural, and language barriers [43]. CHCs could strengthen their contacts with migrant patients by providing service in multiple ways, such as online consultations during off-work hours. Such changes would improve scores on both the NCQA-PCMH and PCAT.

Age might affect how CHC-provided PHC service are perceived. As demonstrated by our PCAT score results, stronger associations were observed among older migrants. Some studies [44, 45] found that elderly individuals use more PHC service than their younger counterparts. As such, older patients might be more sensitive to the effects of PHC service quality. Older adults are vulnerable and require considerable PHC service; thus, their health equity is a national priority. We extend this focus to include the population of elderly *migrant* patients. CHCs should focus on older migrant patients to improve PHC quality, in agreement with the initiative of developing elderly-friendly communities in China, considered a “rapidly aging country” [46].

Although objective in quality assessment, our study had some limitations which warrant consideration. First, our data were obtained from a cross-sectional study, so we cannot infer a temporal association between process and outcomes. Second, the use of self-reported questionnaires is subject to recall bias which could have affected the between-group differences we observed. Finally, the sample size was limited because only ten CHCs participated. Multicentre studies are needed to achieve greater improvements in migrants' experiences of primary health care and health equity. And experiments with health care interventions are needed to validate the relationship between the quality of CHC service and the experiences of migrants. Further discussion with different results from different countries and regions is also necessary to expand the application of PCMH domestically and globally.

## Conclusion

Our results suggest an association between CHC healthcare service quality, as determined by NCQA-PCMH, and migrant patients’ PHC experiences, as assessed by the PCAT. Age acted as a potential modifying factor. These results further indicated that to improve migrant patients’ experiences and health equity, policymakers should base CHC improvement efforts on NCQA-PCMH dimensions considering the unique needs of vulnerable groups, such as migrant patients and the elderly.

## Supplementary Information

**Additional file 1**: **Figure S1.** Study participant and center sampling strategy. **Table S1.** CHC service quality, as determined by the NCQA-PCMH. **Table S2.** the relevant items of NCQA-PCMH and PCAT. 

## Data Availability

All data generated or analysed during this study are included in this published article and its supplementary information files.

## Abbreviations

CHCs: Community health centres
NCQA-PCMH: National Committee for Quality Assurance Patient-Centred Medical Home
PCAT: Primary Care Assessment Tools
PHC: Primary healthcare

## References

[1] IOM. World Migration Report 2022. 2022. https://worldmigrationreport.iom.int/wmr-2022-interactive/. Accessed 6 July 2022.

[2] China NBoSotPsRo. Release of the Main Data of the Seventh National Census of China. 2021. http://www.gov.cn/guoqing/2021-05/13/content_5606149.htm. Accessed 1 July 2022.

[3] Hosseinpoor AR, Bergen N, Schlotheuber A (2015). Promoting health equity: WHO health inequality monitoring at global and national levels. Glob Health Action.

[4] McMichael C, Healy J (2017). Health equity and migrants in the greater mekong subregion. Glob Health Action..

[5] Bureau GS. The Release of the Main Data of the Seventh National Census of Guangzhou. 2021. http://tjj.gz.gov.cn/tjgb/glpcgb/content/post_7286026.html. Accessed 8 July 2022.

[6] Bureau FS. The Release of the Main Data of the Seventh National Census of Foshan. . 2021. http://www.foshan.gov.cn/gzjg/stjj/ztzl_1110965/tjgb_1110961/content/post_4804505.html. Accessed 8 July 2022.

[7] Chen SQ, Chen YY, Feng ZC, Chen X, Wang Z, Zhu JF, Jin J (2020). Barriers of effective health insurance coverage for rural-to-urban migrant workers in China: a systematic review and policy gap analysis. BMC Public Health..

[8] Shao S, Wang MR, Jin GH, Zhao YL, Lu XQ, Du J (2018). Analysis of health service utilization of migrants in Beijing using Anderson health service utilization model. BMC Health Serv Res..

[9] Mou J, Griffiths SM, Fong HF, Dawes MG (2015). Defining migration and its health impact in China. Public Health.

[10] Rawaf S, De Maeseneer J, Starfield B (2008). From Alma-Ata to Almaty: a new start for primary health care. Lancet.

[11] China GOotSCotPsRo. The Outline of the 14th Five-Year Plan (2021–2025) for National Health. 2022. http://www.gov.cn/zhengce/content/2022-05/20/content_5691424.htm. Accessed 30 May 2022.

[12] China tSCotPsRo. The Plan of Health China 2030. 2016. http://www.gov.cn/zhengce/2016-10/25/content_5124174.htm. Accessed 10 June 2020.

[13] Yip WCM, Hsiao WC, Chen W, Hu SL, Ma J, Maynard A (2012). Early appraisal of China's huge and complex health-care reforms. Lancet.

[14] Wu J, Liu R, Shi L, Zheng L, He N, Hu R (2022). Association between resident status and patients' experiences of primary care: a cross-sectional study in the Greater Bay Area. China BMJ Open.

[15] Madden H, Harris J, Blickem C, Harrison R, Timpson H (2017). "Always paracetamol, they give them paracetamol for everything": a qualitative study examining Eastern European migrants' experiences of the UK health service. BMC Health Serv Res..

[16] Li HT, Chung RYN, Wei XL, Mou J, Wong SYS, Wong MCS (2014). Comparison of perceived quality amongst migrant and local patients using primary health care delivered by community health centres in Shenzhen. China. BMC Fam Pract..

[17] Goth UGS, Berg JE (2011). Migrant participation in Norwegian health care.  A qualitative study using key informants.. Eur J Gen Pract.

[18] Lindenmeyer A, Redwood S, Griffith L, Teladia Z, Phillimore J. Experiences of primary care professionals providing healthcare to recently arrived migrants: a qualitative study. BMJ Open. 2016;6:e012561 10.1136/bmjopen-2016-012561 PMC5051449 27660320

[19] Zeng J, Shi L, Zou X, Chen W, Ling L (2015). Rural-to-Urban migrants' experiences with primary care under different types of medical institutions in Guangzhou. China PLoS One.

[20] Donabedian A (1969). Quality of care: problems of measurement. II. Some issues in evaluating the quality of nursing care. Am J Public Health Nations Health.

[21] Liu R, Shi L, Meng Y, He N, Wu J, Yan X, Hu R (2021). The institutional primary healthcare service quality and patients' experiences in Chinese community health centres: results from the Greater Bay Area study. China Int J Equity Health.

[22] Hu R, Liao Y, Du Z, Hao Y, Liang H, Shi L (2016). Types of health care facilities and the quality of primary care: a study of characteristics and experiences of Chinese patients in Guangdong Province. China BMC Health Serv Res.

[23] Harzheim E, Pinto LF, D'Avila OP, Hauser L. Measuring the quality of primary care in national health surveys: Lessons from Brazil. Afr J Prim Health Care Fam Med. 2020;12(1):a2251. 10.4102/phcfm.v12i1.2251 PMC7061225 32129645

[24] Hoa NT, Tam NM, Derese A, Markuns JF, Peersman W. Patient experiences of primary care quality amongst different types of health care facilities in central Vietnam. BMC Health Serv Res. 2019;19:275. 10.1186/s12913-019-4089-y PMC6498623 31046750

[25] Jin H, Wang ZX, Shi LY, Chen C, Huo YY, Huang WQ, Zhang Y, Lu Y, Ge XH, Shi JW, Yu DH. Multimorbid Patient Experiences With Primary Care at Community Health Centers in Shanghai. China Front Public Health. 2021;9:606188. 10.3389/fpubh.2021.606188 PMC8218628 34169053

[26] Kijima T, Matsushita A, Akai K, Hamano T, Takahashi S, Fujiwara K, Fujiwara Y, Sato M, Nabika T, Sundquist K, et al. Patient satisfaction and loyalty in Japanese primary care: a cross-sectional study. BMC Health Serv Res. 2021;21:274. 10.1186/s12913-021-06276-9 PMC7992825 33766027

[27] Cho Y, Chung H, Joo H, Park HJ, Joh HK, Kim JW, Lee JK. Comparison of patient perceptions of primary care quality across healthcare facilities in Korea: A cross-sectional study. PLoS One. 2020;15(3):e0230034. 10.1371/journal.pone.0230034 PMC7064208 32155199

[28] NCQA. About NCQA. 2022. https://www.ncqa.org/about-ncqa/. Accessed 18 July 2022.

[29] Jabbarpour Y. The Impact of Primary Care Practice Transformation on Cost , Quality , and Utilization. 2017. https://www.pcpcc.org/sites/default/files/resources/pcmh_evidence_report_08-1-17%20FINAL.pdf. Accessed 12 August 2022.

[30] Store N. PATIENT-CENTERED MEDICAL HOME (PCMH). 2022. https://store.ncqa.org/index.php/recognition/patient-centered-medical-home-pcmh.html. Accessed 18 July 2022.

[31] NCQA. Patient-Centered Medical Home (PCMH). 2022. https://www.ncqa.org/programs/health-care-providers-practices/patient-centered-medical-home-pcmh/. Accessed 17 July 2022.

[32] Owolabi O, Zhang ZZ, Wei XL, Yang N, Li HT, Wong SYS, Wong MCS, Yip W, Griffiths SM (2013). Patients' socioeconomic status and their evaluations of primary care in Hong Kong. BMC Health Serv Res..

[33] Sung NJ, Markuns JF, Park KH, Kim K, Lee H, Lee JH (2013). Higher quality primary care is associated with good self-rated health status. Fam Pract.

[34] Macinko J, Mullachery PH, Primary care experiences among Brazilian adults: Cross-sectional evidence from the,  (2019). National Health Survey. PLoS ONE.

[35] Wang HHX, Wong SYS, Wong MCS, Wang JJ, Wei XL, Li DKT, Tang JL, Griffiths SM (2015). Attributes of primary care in community health centres in China and implications for equitable care: a cross-sectional measurement of patients' experiences. Qjm-an Int J Med.

[36] Donabedian A (1988). The quality of care. How can it be assessed?. Jama.

[37] Stange KC, Nutting PA, Miller WL, Jaen CR, Crabtree BF, Flocke SA, Gill JM (2010). Defining and Measuring the Patient-Centered Medical Home. J Gen Intern Med.

[38] Solberg LI, Asche SE, Fontaine P, Flottemesch TJ, Anderson LH (2011). Trends in quality during medical home transformation. Ann Fam Med.

[39] Haggerty JL, Reid RJ, Freeman GK, Starfield BH, Adair CE, McKendry R (2003). Continuity of care: a multidisciplinary review. BMJ.

[40] Shi L, Patil VP, Leung W, Zheng QM (2022). Willingness to use and satisfaction of primary care services among locals and migrants in Shenzhen, China. Health Soc Care Community.

[41] Henry TL, Britz JB, Louis JS, Bruno R, Oronce CIA, Georgeson A, Ragunanthan B, Green MM, Doshi N, Huffstetler AN (2022). Health equity: the only path forward for primary care. Ann Fam Med.

[42] Eissa A, Rowe R, Pinto A, Okoli GN, Campbell KM, Washington JC, Rodriguez JE (2022). Implementing high-quality primary care through a health equity lens. Ann Fam Med.

[43] Shi L (2012). The impact of primary care: a focused review. Scientifica (Cairo).

[44] Lin Y, Chu C, Chen Q, Xiao J, Wan C (2020). Factors influencing utilization of primary health care by elderly internal migrants in China: the role of social contacts. BMC Public Health.

[45] Song X, Zou G, Chen W, Han S, Zou X, Ling L (2017). Health service utilisation of rural-to-urban migrants in Guangzhou, China: does employment status matter?. Trop Med Int Health.

[46] China tSCotPsRo. Notice on the establishment of a model national elderly-friendly community. 2020. http://www.gov.cn/zhengce/zhengceku/2020-12/14/content_5569385.htm. Accessed 20 Oct 2022.

