# Supplementary material for: The quality of migrant patients’ primary healthcare experiences and patient-centered medical home achievement by community health centers: results from the China greater bay area study
Source: Int J Equity Health. 2023 Jun 7;22:114. doi: 10.1186/s12939-023-01929-z (PMC10245519; doi:10.1186/s12939-023-01929-z)
Supplement: Supplementary file 1 — Additional file 1: Figure S1. Study participant and center sampling strategy. Table S1. CHC service quality, as determined by the NCQA-PCMH. Table S2. the relevant items of NCQA-PCMH and PCAT. [file 12939_2023_1929_MOESM1_ESM.pdf]

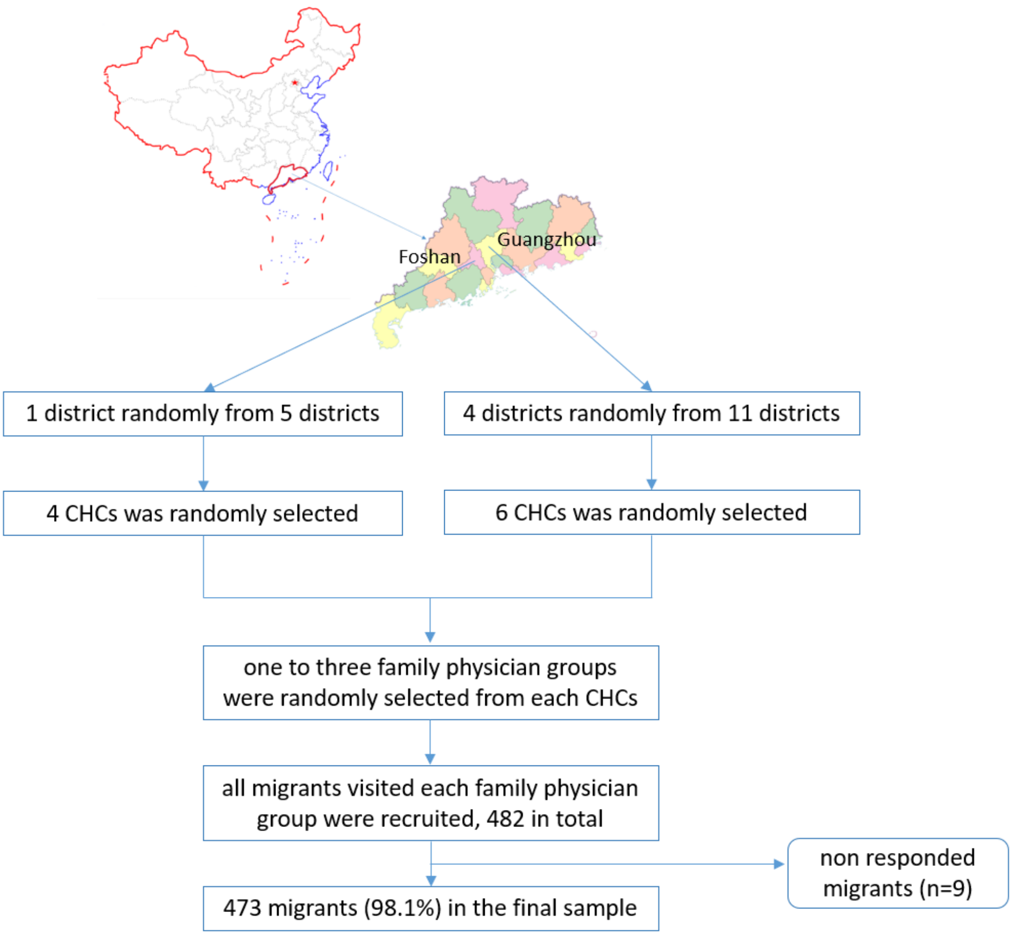

Figure S1. Study participant and center sampling strategy

Table S1. CHC service quality, as determined by the NCQA-PCMH

	PCMH1	PCMH2	PCMH3	PCMH4	PCMH5	PCMH6	Total	Level
Guangzhou								
LH	6.5	5.9	13.3	10	12	11.8	59	1
LD	8	7.1	12	11.3	1.5	1.5	41	1
HL	7.3	6.9	13.8	12	0	10.5	51	1
JH	7.6	7.9	18	8.3	13.5	15.3	71	2
HHG	7	5.3	12.8	19	10.5	18.3	73	2
SY	9.5	11.4	18	14	16.5	17.3	87	3
Foshan								
FFCH	10	11.4	19	18	15	11.5	85	3
CHZH	7.9	5.4	10.5	1.3	3	2.8	31	1
YH	5.3	3.6	10.3	6.5	1.5	0.8	28	1
NTFPH	2.6	9.1	17.5	10.3	12	7.3	59	1
Mean±SD	7.2±2.0	7.4±2.5	14.5±3.2	11.1±5.0	8.6±6.0	9.7±6.1	58±19.4	

LH, Linhua; LD, Liede; HL, Hualin; JH, Jianghai; HHG, HuangHuagang; SY, Shayuan; FFCH, Foshan Fosun-Chancheng Hospital; CHZH, Chancheng High-tech Zone Hospital; YH, Yong’an Hospital; NTFPH, Nanzhuang Town the First People’s Hospital in Chancheng.

CHCs, community health centers.

Table S2 the relevant items of NCQA-PCMH and PCAT

Contact-access	PCMH	PCMH1. Patient-Centered Access	A1. Providing same-day appointments for routine and urgent care.
			A4. Availability of appointments.
	PCAT	C First Contact-Access	C3. When your PCP is open and you get sick, would someone from there see you the same day?
Outside the clinic	PCMH	PCMH1. Patient-Centered Access	A3. Providing alternative types of clinical encounters.
			B2. Providing timely clinical advice by telephone.
			C2. The capability to view, download or transmit their health information to a third party.
	PCAT	C First Contact-Access	C4. When your PCP is open, can you get advice quickly over the phone if you need it?
		D Ongoing Care	D4. If you have a question, can you call and talk to the doctor or nurse who knows you best?
Case information	PCMH	PCMH1. Patient-Centered Access	B1. Providing continuity of medical record information for care and advice when office is closed.

preservation	PCAT	F Coordination (Information Systems)	F3. When you go to your doctor, is your medical record always available?
Patients themselves are aware of personal health information	PCMH	PCMH1. Patient-Centered Access	C1. More than 50 percent of patients have timely access to their health information.
			C3. Clinical summaries are provided to patients/families/caregivers upon request.
		PCMH3. Population Health Management	Element A - Patient Information
			Element B - Clinical Data
	PCAT	F Coordination (Information Systems)	F2. Could you look at your medical record if you wanted to?
			F3. When you go to your PCP, is your medical record always available?
Personal continuity care	PCMH	PCMH2. Team-Based Care	A1. Assisting patients/families to select a personal clinician and documenting the selection in practice records.
	PCAT	D Ongoing Care	D1. When you go to your PCP's, are you taken care of by the same doctor or nurse each time?

Family-centeredness	PCMH	PCMH2. Team-Based Care	A4. Collaborating with the patient/family to develop/implement a written care plan for transitioning from pediatric care to adult care.
		PCMH4. Care Management and Support	B5. Is provided in writing to the patient/family/caregiver.
	PCAT	I Family-Centeredness	I1. Does your PCP ask you about your ideas and opinions when planning treatment and care for you or a family member?
Referral Coordination	PCMH	PCMH2. Team-Based Care	B1. The practice is responsible for coordinating patient care across multiple settings.
			B8. Instructions on transferring records to the practice, including a point of contact at the practice.
		PCMH5. Care Coordination and Care Transitions	B5. Gives the consultant or specialist the clinical question, the required timing and the type of referral.
			B7. Has the capacity for electronic exchange of key clinical information and provides an electronic summary of care record to another provider for more than 10 percent of referrals.

			B9. Documents co-management arrangements in the patient medical record.
			B10. Asks patients/families about self-referrals and requesting reports from clinicians.
			C2. Shares clinical information with admitting hospitals and emergency departments.
			C3. Consistently obtains patient discharge summaries from the hospital and other facilities.
			C7. Exchanges key clinical information with facilities and provides an electronic summary-of-care record to another care facility for more than 10 percent of patient transitions of care.
	PCAT	E Coordination	E8. Did your PCP discuss with you different places you could have gone to get help with that problem?
			E9. Did your PCP or someone working with your PCP help you make the appointment for that visit?

			E10. Did your PCP write down any information for the specialist about the reason for the visit?
			E12. After you went to the specialist or special service, did your PCP talk with you about what happened at the visit?
		F Coordination (Information Systems)	F1. When you go to your PCP, do you bring any of your own medical records, such as shot records or reports of medical care you had in the past?
Language services	PCMH	PCMH2. Team-Based Care	ELEMENT C - Culturally and Linguistically Appropriate Services
	PCAT	K Culturally Competent	K2. Would you recommend your PCP to someone who does not speak English well?
Comprehensive health care	PCMH	PCMH2. Team-Based Care	D6Training and assigning members of the care team to support patients/families/caregivers in self-management, self-efficacy and behavior change.
		PCMH4. Care Management and	A1. Behavioral health conditions.

		Support	B1. Incorporates patient preferences and functional/lifestyle goals.
			E6. Maintains a current resource list on five topics or key community service areas of importance to the patient population including services offered outside the practice and its affiliates.
	PCAT	D Ongoing Care	D7. Does your PCP know you very well as a person, rather than as someone with a medical problem?
			D9. Does your PCP know what problems are most important to you?
		J Community orientation	J1. Does anyone at your PCP's office ever make home visits?
Patient engagement	PCMH	PCMH2. Team-Based Care	D10. Involving patients/families/caregivers in quality improvement activities or on the practice advisory council.
	PCAT	J Community orientation	J3. Does your PCP get opinions and ideas from people that will help to provide better health care?
Drug use follow-up	PCMH	PCMH3. Population Health Management	D5. Medication monitoring or alert.

		PCMH4. Care Management and Support	ELEMENT C - Medication Management
			C1. Reviews and reconciles medications for more than 50 percent of patients received from care transitions.
			C2. Reviews and reconciles medications with patients/families for more than 80 percent of care transitions.
			C3. Provides information about new prescriptions to more than 80 percent of patients/families/caregivers.
			C4. Assesses understanding of medications for more than 50 percent of patients/families/caregivers, and dates the assessment.
			C6. Assesses response to medications and barriers to adherence for more than 50 percent of patients, and dates the assessment.
	PCAT	H Comprehensiveness (Services Provided)	H7. Checking on and discussing the medications you are taking
Immunization	PCMH	PCMH3. Population Health Management	C1. Age- and-gender appropriate immunizations and screenings.

	PCAT	G Comprehensiveness (Services Available)	G2. Immunizations (shots)

PCAT: the Primary Care Assessment Tools; PCMH: Patient-Center Medical Home.
